# Co-expression of *G2-EPSPS* and glyphosate acetyltransferase *GAT* genes conferring high tolerance to glyphosate in soybean

**DOI:** 10.3389/fpls.2015.00847

**Published:** 2015-10-15

**Authors:** Bingfu Guo, Yong Guo, Huilong Hong, Longguo Jin, Lijuan Zhang, Ru-Zhen Chang, Wei Lu, Min Lin, Li-Juan Qiu

**Affiliations:** ^1^The National Key Facility for Crop Gene Resources and Genetic Improvement, Institute of Crop Science, Chinese Academy of Agricultural Sciences, Beijing, China; ^2^College of Agriculture, Northeast Agricultural University, Harbin, China; ^3^Biotechnology Research Institute, Chinese Academy of Agricultural Sciences, Beijing, China

**Keywords:** transgenic soybean, glyphosate tolerance, *G2-EPSPS*, *GAT*, chlorophyll content, shikimic acid

## Abstract

Glyphosate is a widely used non-selective herbicide with broad spectrum of weed control around the world. At present, most of the commercial glyphosate tolerant soybeans utilize glyphosate tolerant gene *CP4-EPSPS* or glyphosate acetyltransferase gene *GAT* separately. In this study, both glyphosate tolerant gene *G2-EPSPS* and glyphosate degraded gene *GAT* were co-transferred into soybean and transgenic plants showed high tolerance to glyphosate. Molecular analysis including PCR, Sothern blot, qRT-PCR, and Western blot revealed that target genes have been integrated into genome and expressed effectively at both mRNA and protein levels. Furthermore, the glyphosate tolerance analysis showed that no typical symptom was observed when compared with a glyphosate tolerant line HJ06-698 derived from GR1 transgenic soybean even at fourfold labeled rate of Roundup. Chlorophyll and shikimic acid content analysis of transgenic plant also revealed that these two indexes were not significantly altered after glyphosate application. These results indicated that co-expression of *G2-EPSPS* and *GAT* conferred high tolerance to the herbicide glyphosate in soybean. Therefore, combination of tolerant and degraded genes provides a new strategy for developing glyphosate tolerant transgenic crops.

## Introduction

Soybean [*Glycine max* (L.) Merr.], an important and most frequently cultivated grain legume in the worldwide, is the economic source of both vegetable oil and protein meal with about 20% oil and 40% protein content in its seeds (Hartman et al., 2011). Among them, about 95% of the oil fraction is consumed as edible oil and about 98% of soybean meal is used in livestock and aquaculture feeds due to its high protein level (Liu et al., 2008). Soybean is unique among crops in that it supplies protein nearly equal in quality to that of animal sources but with less saturated fat and no cholesterol (Young, 1991). In addition, it plays an important role in crop diversification and benefits to other crops due to its capacity for addition of atmospheric nitrogen to the soil during crop rotation (Singh et al., 2010).

Weed is defined as plant whose undesirable qualities outweigh their good points (Randall, 1997). Weeds are troublesome in many ways, in which they mainly reduce crop yield by competing for light, water, soil nutrients, and space. Weeds are so common on cropland that their economic impact on crop losses and control costs has been estimated for different crops. In general, about 15% of soybean seed yield was reduced due to the harm of weeds (Yao, 2009). Therefore, weed control becomes a significant process for ensuring high and stable yield of crops. There are many methods available to control weeds, including preventative, cultural, mechanical, biological, and chemical weed control. Since chemical method is very economic, highly efficient and easy to operate, it has been an important strategy in modern weed management in crop field (Zhang, 2011). Based on expenditures, about 30 and 13% of global agrochemical sales are committed to the purchase of selective and non-selective herbicide respectively (Edwards and Hannah, 2014).

Along with the extensive use of herbicides, weeds were becoming widely resistant to commonly used selective herbicides. The ability to use glyphosate (*N*-phosphonomethylglycine) in glyphosate-resistant (GR) crops made weed management easy, efficient, economical and environmentally compatible (Green, 2012). Glyphosate’s mode of action is non-selectively inhibiting the plant enzyme 5-enolpyruvylshikimate-3-phosphate synthase (EPSPS) which involved in the biosynthesis pathway for aromatic amino acid (Padgette et al., 1995; Tan et al., 2006). Because of this mode of action, it is effective on actively growing plants, whether they are crops or weeds. The ability to use biotechnology to make glyphosate tolerant transgenic crops allows farmers to use glyphosate as a postemergence herbicide against both broadleaf and cereal weeds (Dill et al., 2008).

Up to now, two strategies for achieving glyphosate tolerance have been successfully applied in GM plants. The first one is overproduction of target enzyme EPSPS, such as expression of exogenous *CP4-EPSPS* (Meilan et al., 2002) or mutant *EPSPS* in transgenic plants (Tian et al., 2011). One of the disadvantages of this strategy is that glyphosate remains to be accumulated in plant tissues and decrease crop yield by interfering with the development of reproductive tissues (Pline et al., 2002). For example, although the labeled rate for glyphosate application in GR soybeans is varies from 600 to 1200 g a.e.ha^–1^ and the tolerant level could reach to threefolds of labeled rate (2400 g a.e. ha^–1^) at some growth stages, some physiological indexes including photosynthesis, nutrient accumulation, and nodulation in some cultivars was still reduced after receiving increasing glyphosate rates or applications at later growth stages (Gazziero et al., 2008; Zobiole et al., 2012). Meanwhile, the typical symptom, known as “yellow flashing,” was observed in both GR1 and GR2 soybeans (Zobiole et al., 2012). This symptom is attributed to the decrease of chlorophyll content, accumulation of the primary phytotoxic metabolite or the formation of insoluble glyphosate-metal complexes (Campbell et al., 1976; Reddy et al., 2004; Zobiole et al., 2012). The second strategy is removal of herbicidal residue after detoxification it by glyphosate oxidoreductase (*GOX*) gene (Pedotti et al., 2009) or glyphosate N-acetyltransferase (*GAT*) gene (Castle et al., 2004; Siehl et al., 2005). GOX is an enzyme which can degrade glyphosate and converting it to glyoxylate and aminomethylphosphonic acid (AMPA). Although AMPA is less phytotoxic than glyphosate to plant, it could still induce “yellow flashing” (Ding et al., 2011). Another enzyme GAT could degrade glyphosate residues by catalyzing acetylation of glyphosate. Therefore, overexpression of the *GOX* or *GAT* gene can also result in a relatively high level of glyphosate tolerance in different crops (Green et al., 2008; Hadi et al., 2012). Dun et al. (2014) compared the glyphosate tolerance of transgenic tobacco which expressed *GAT* and *EPSPS* alone or combination and the results suggested that co-expression of them showed the highest tolerance to glyphosate, providing a new method to develop high glyphosate tolerant crops by combination of different strategies.

In this study, both *G2-EPSPS* and *GAT* genes were co-transferred into soybean by *Agrobacterium*-mediated transformation method. Molecular and phenotype analysis revealed that co-expression of these two genes conferred high glyphosate tolerance. Especially, the high tolerant line of transgenic soybean did not show typical symptom of shikimic acid accumulation, “yellow flashing” and chlorophyll content reduction when compared with a GR1 cultivar HJ06-698. These results suggested that the new developed transgenic line with high tolerance to glyphosate could be used for soybean breeding.

## Materials and Methods

### Plant Materials and Vector Information

**FIGURE 1:**
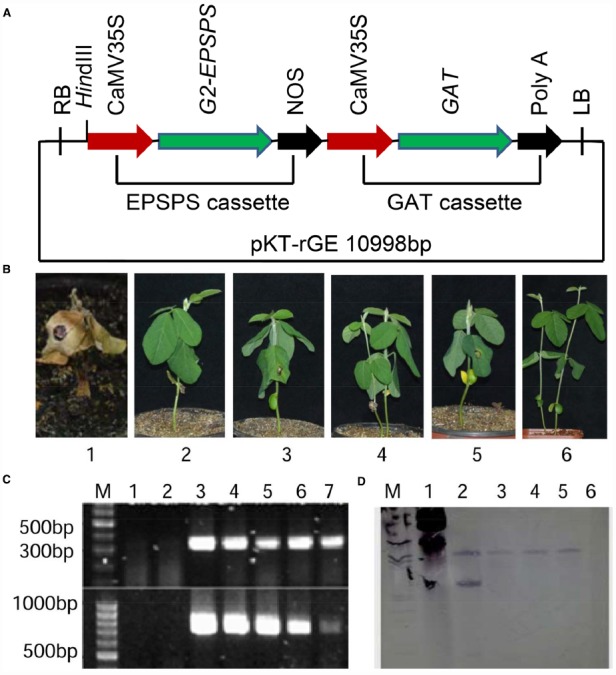
**Transformation vector and identification of transgenic T_0_ lines. (A)** Schematic representation of plant transformation vector (pKT-rGE) showing the two expression cassettes for *G2-EPSPS* and *GAT* genes. **(B)** Tolerant level of T_0_ putative transgenic plants identified by pipette spotting of 1 μl Roundup (0.3 mg a.e.μl^–1^ glyphosate). 1: non-transgenic wild type soybean treated with Roundup; 2: non-transgenic soybean treated with water; 3–5: four putative transgenic lines treated with Roundup; 6: glyphosate tolerant soybean HJ06-698 treated with Roundup. **(C)** The glyphosate tolerant transgenic plants were confirmed by PCR analysis. M: 100 bp DNA marker; 1: negative control of non-transgenic receptor; 2: negative control of water; 3: positive control of pKT-rGE vector; 4–7: four putative transgenic plants. **(D)** Southern blot analysis of four glyphosate tolerance putative transgenic lines. M: DIG marker; 1: positive control of pKT-rGE Vector; 2–5: DNA samples of four putative transgenic plants digested with *Hin*dIII; 6: DNA sample of non-transgenic soybean receptor was used as the negative control.

Soybean cultivars Jack and ZH10, and the binary vector pKT-rGE (Beijing Weiming Kaituo Biotech Co. Ltd, China), were used for transformation. Glyphosate tolerant soybean HJ06-698, a backcross cultivar derived from GTS40-3-2 (GR1 soybean), was used as positive control for glyphosate tolerance analysis. The pKT-rGE contains a *GAT* gene from *Bacillus licheniformis* and glyphosate tolerant gene (*G2-EPSPS*) from *Pseudomonas fluorescens* G2 driven by two CaMV 35S promoters separately (Figure 1A).

### Genetic Transformation

The plant expression vector pKT-rGE was transformed into *Agrobacterium* strain Ag10. The *Agrobacterium*-mediated soybean cotyledonary node genetic transformation method was performed according to Guo et al. (2015) with some modification. Briefly, seedlings geminated from sterilized mature seeds on germination medium at 24°C for 5 days were used for preparing cotyledonary node explants. After the explants were infected with *Agrobacterium* suspensions supplemented with 0.02% Silwet L-77 and sonicated at 35 kHz for 2 s, they were placed on co-culture medium and incubated at 24°C in dark for 3 days. In order to improve the infection efficiency of *Agrobacterium* and inhibit the browning of explants, the co-culture medium was supplemented with 40 μmol L^–1^ AS, 1 μmol L^–1^ DTT, 1 μmol L^–1^ Na_2_S_2_O_3_, and 8.8 μmol L^–1^L-Cysteine. The glyphosate (Sigma-Aldrich, USA) with final concentrations of 15 mg a.e.L^–1^ and 5 mg a.e.L^–1^ were used for plant selection in the medium for bud induction and shoot elongation respectively. Putative transgenic soybean plants were selected from resistant bud induction, shoot elongation and rooting.

### Genomic DNA Extraction and PCR Amplification

Genomic DNA was isolated from young leaves of putative transgenic plants using the modified CTAB method (Murray and Thompson, 1980). A 743 bp fragment of *G2*-*EPSPS* and 338 bp of *GAT* were amplified using gene-specific primers (Table 1). The PCR reactions were carried out in a total volume of 20 μl using PTC-200 Thermocycler (MJ Research/Bio-Rad, USA) with the PCR reaction cycles as follows: 1 cycle (94°C, 4 min), 36 cycles (94°C, 30 s; 60°C, 30 s; 72°C, 45 s), and a final extension step (72°C, 10 min). PCR products were analyzed on 1% agarose gels by electrophoresis.

**TABLE 1:** **Primers used in this study**

**Primer Name**	**Sequence (5′-3′)**	**Fragment Length (bp)**
G2EP-F	5′-ACCAGGAGCCTTGTACCTTGAG-3′	743
G2EP-R	5′-ATCGGGTTCGATCAGGTAATC-3′	
GAT-F	5′- CTCAGACCAAACCAGCCGATAG -3′	338
GAT-R	5′- GTAGTAGCCTGAGGCGGATGTC C-3′	
ACT-RTF	5′- CGGTGGTTCTATCTTGGCATC -3′	142
ACT-RTR	5′- GTCTTTCGCTTCAATAACCCTA-3′	
CP4-RTF	5′- GCAAATCCTCTGGCCTTTCC-3′	146
CP4-RTR	5′- CTTGCCCGTATTGATGACGTC -3′	
G2EP-RTF	5′- CGACATTACTTCCATCACAAGCA-3′	150
G2EP-RTR	5′- CCCGAATCATCAGGCAAACA-3′	
GAT-RTF	5′- GCGGACTTGCTTTGGTGTAAT-3′	168
GAT-RTR	5′-AACTACTCACACATTATTATGGAGAAACT-3′	

### Southern Blotting

Randomly-selected transgenic T_0_ plants confirmed by glyphosate tolerance and PCR analysis were analyzed by Southern blotting to check the integration of the exogenous genes. 40–60 μg genomic DNA (gDNA) from transgenic plants and receptor plants (negative control) were digested with restriction enzyme. To detect specific fragment for each T-DNA insertion, the restriction enzyme (*Hin*dIII) with only one cleave site in T-DNA region of pKT-rGE was used for digestion. gDNA after digestion were separated by agarose gel electrophoresis and was transferred to a nylon membrane by rapid downward transfer systems (Whatman/Schleicher & Schuell) after electrophoresis. A Dig-dUTP probe for *GAT* coding sequence was amplified from pKT-rGE plasmid using DIG Probe Synthesis Kit (Mylab Corporation, China) with gene specific primers GAT-F/R (Table 1). The vector was also digested with *Hind*III and an 11.0 kb fragment containing the *GAT* coding sequence could be hybridized by the probe. After hybridization, the bands on the membrane were detected with the chemi-color substrate NBT/BCIP (Mylab Corporation, China).

### Semi-quantitative RT-PCR and qRT-PCR

Total RNA extracted from leaves of four transgenic lines with RNA extraction kit (TRIzol reagent, Invitrogen, Inc.) was used for semi-quantitative RT-PCR. For qRT-PCR, RNA was isolated from different tissues (young leaves, mature leaves, stem, flower, and seed) of Line 1 or leaves of Line 1 and HJ06-698 treated with 900 g a.e. ha^–1^ glyphosate at different time points (0, 6, 12, 24, 48, 72, 120, and 192 h). The cDNA was synthesized using the PrimerScript^TM^ II 1st strand cDNA synthesis kit (Takara, Inc.,). Specific primers for *G2-EPSPS* and *GAT* genes were used for semi-quantitative RT-PCR and qRT-PCR (Table 1). The expression levels of different genes were calculated using 2^–ΔΔt^ method and standardized to the constitutive expression level of *Actin* (Kenneth and Schmittgen, 2001). At least three biological replications were carried out for each sample.

### Western Blotting

About 0.1g samples of leaves, roots and stem were ground in liquid nitrogen and total protein were extracted from four transgenic lines with plant protein extraction kit (Cwbiotech, Inc.,) in accordance with the manufacturer’s instructions. The protein concentrations were measured using the Bradford method (Bradford, 1976). Equal amounts of proteins (35 μg) were boiled at 95°C for 5 min with 1/4 volume of 5 × sodium dodecylsulfate loading buffer and separated on 10% gels using SDS-PAGE. Heat shock protein (HSP) was used as the reference protein for normalization of the protein level. The proteins were then transferred electrophoretically to a PVDF membrane with transfer buffer (10 mmol L^–1^ glycine, 25 mmol L^–1^ Tris, and 10% (V/V) methanol, pH8.2) and then washed with 10 ml TBS for 5 min. After blocking with 5% dried skimmed milk (diluted by TTBS) for 1 h at room temperature, the membrane was incubated with antiserum (1:5000 dilution) for 1 h and then washed with 100–200 ml TTBS for three times (5 min each). Then, the membrane was incubated with 1:5000 diluted anti-rabbit IgG for 1h at room temperature and washed with 100–200 ml TTBS for three times (5 min each). The reaction was visualized with ECL chemi-color substrate.

### Glyphosate Tolerance Analysis

Commercially formulated isopropylamine salt of glyphosate with the rate of 300 g a.e.L^–1^ (Roundup, Monsanto Company) was used for glyphosate tolerance analysis. The labeled rate for glyphosate application in GR soybeans varies from 600 to 1200 g a.e.ha^–1^ (Gazziero et al., 2008) and the application label rate for the crop production region of our study site is 900 g a.e.ha^–1^ according to the manufacture’s manual. Two methods including pipette spotting and spraying methods were used to analyze glyphosate tolerance in transgenic soybean plants. The first method was carried out for T_0_ plants and the other one was for T_2_ plants. For the pipette spotting method, 1 μl of Roundup (0.3 mg a.e.μl^–1^ glyphosate) was pipette spotted in young leaves after the rooting plantlets were transferred into pot culture. Glyphosate tolerance analysis of transgenic T_2_ plants was performed according to Zobiole et al. (2012) with some modification. When the first trifoliolate leaves of transgenic T_2_ plants were fully expanded, plants were spraying with Roundup at rates of 900, 1800, 2700, and 3600 g a.e.ha^–1^. The rates 2700 and 3600 g a.e.ha^–1^ was selected to represent the “worst-case scenario” to promote soybean injury. The phytotoxicity symptoms and survival number of glyphosate tolerance plants were investigated after 2 weeks.

### Chlorophyll Content Analysis

Chlorophyll content was measured according to Richardson et al. (2002). Thirty-day old plants of transgenic line 1, HJ06-698, ZH10, and MD12 were spraying with Roundup at labeled rate of 900 g a.e.ha^–1^ using water as negative control. The SPAD value of leaves from different soybean plants were measured with a SPAD-502 Plus chlorophyll measuring instrument at 0, 6, 12, 24, 48, 72, and 120 h after treatments. Absorption at 650 and 940 nm was used to estimate chlorophyll content. At least eight biological replications were performed for each treatment and the data was analyzed using SPSS 18.0 and Excel. The one way ANOVA was used for significant difference analysis.

### Shikimic Acid Content Analysis

The content of shikimic acid was measured according to Gao et al. (2013). Briefly, 30-day old soybeans of transgenic line 1, HJ06-698, ZH10, and MD12 were spraying with the Roundup at labeled rate of 900 g a.e.ha^–1^. After the application of glyphosate, 0.2 g leaves added with 1 ml of 0.25 mol L^–1^ HCl were grounded into homogenate suspension rapidly on ice. After the mixture was centrifuged at 14,000 rpm for 15 min (4°C), 100 μl of supernatant was taken and mixed with 2 ml of 0.1% periodic acid to oxidize shikimic acid for 3 h. Then, the reaction solution was mixed with 2 ml NaOH (0.1 mol/L) and 1.2 ml glycine (0.1 mol/L). Finally, the optical density of the solution was measured at 380 nm. The content of shikimic acid was calculated according to the standard curve plotting by using standard samples at different concentrations. The one way ANOVA was used for significant difference analysis.

## Results

### Transformation and Molecular Analysis of Transgenic Soybean Plants

Soybean plants were transformed with *35S-G2EPSPS/35S-GAT* and a total of 22 independent putative transgenic lines were selected using *Agrobacterium*-mediated transformation method. After the rooting plantlets were transferred into pot culture, the T_0_ plants were initially confirmed by treatment with 1 μl Roundup (0.3 mg a.e.μl^–1^ glyphosate) in young leaves. Two weeks after the treatment, four transgenic soybeans were identified to show high tolerance to glyphosate compared to wild type control (Figure 1B).

In order to confirm the integration of exogenous genes in putative T_0_ transgenic plants, PCR analysis and Southern blot were used for molecular analysis. Expected 743 bp products (for *G2-EPSPS* gene) and 338 bp products (for *GAT* gene) were detected in all four T_0_ putative transgenic plants while no PCR product was detected in the non-transgenic control when using specific primers for both genes (Figure 1C). Moreover, Southern blot analysis of four glyphosate tolerance transgenic lines also suggested that *G2-EPSPS/GAT* were stably integrated into genome of these positive transgenic plants (Figure 1D).

### Expression Analysis of Transgene by Semi-quantitative RT-PCR and Western Blot

**FIGURE 2:**
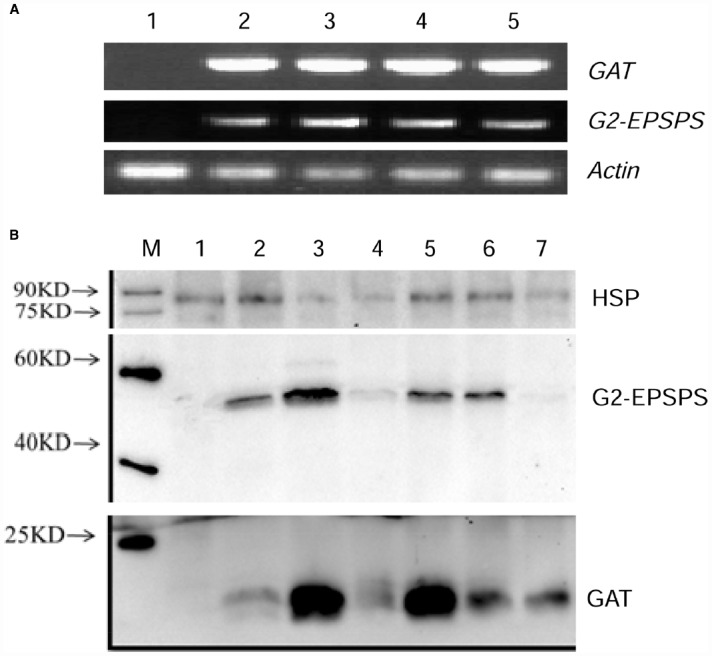
**Expression analysis of four transgenic lines by semi quantitative RT-PCR and western blot. (A)** The expression of exogenous genes in leaves of four transgenic lines was carried out by semi quantitative RT-PCR. 1: negative control of non-transgenic receptor soybean; 2–5: four putative transgenic lines. **(B)** Western blot analysis of four transgenic lines, M: Marker; 1: negative control of non-transgenic soybean; 2–4: roots, leaves and stem of transgenic line 1; 5–7: leaves of transgenic line 2–4.

Semi-quantitative RT-PCR was performed to detect the transcription level of *G2-EPSPS* and *GAT* genes in leaves of four transgenic plants. The transgenic lines showed accumulation of *G2EPSPS/GAT* transcript while the non-transgenic control did not show any specific band, indicating no transcript accumulation in the negative control (Figure 2A).

Western blot was also carried out to quantify the protein accumulation of G2EPSPS/GAT in different transgenic lines. The results revealed that different levels of G2EPSPS/GAT proteins were detected in all transgenic plants (Figure 2B). No protein expression was detected in non-transformed wild type plant. Especially, line 1 and line 2 showed highest protein expression level either for G2EPSPS or GAT and line 1 was selected for further analysis.

### Glyphosate Tolerance Analysis of Transgenic Soybean

**FIGURE 3:**
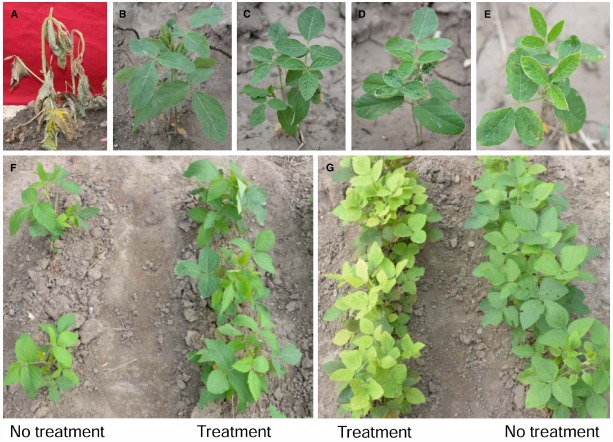
**Phenotype of line 1 after treatment with glyphosate. ZH10 was sprayed with (A)** or without **(B)** Roundup at the rate of 900 g a.e.ha^–1^; Line 1 was sprayed with Roundup at the rate of 900 g a.e.ha^–1^ **(C)**, 1800 g a.e.ha^–1^ **(D)**, and 2700 g a.e.ha^–1^ **(E)**; **(F)** Line 1 was sprayed with or without Roundup at the rate of 3600 g a.e.ha^–1^; **(G)** HJ06-698 was sprayed with or without Roundup at the rate of 3600 g a.e.ha^–1^.

In order to detect the glyphosate tolerance level of transgenic soybean, T_2_ plants of Line 1 were planted in the field and sprayed with Roundup at the doses of 900, 1800, 2700, and 3600 g a.e.ha^–1^ respectively. ZH10 and HJ06-698 were used for non-transgenic and transgenic control. One week after glyphosate application, the leaves of non-transgenic control (ZH10) were shriveled severely and showed clear symptoms compared with no treatment of glyphosate (Figures 3A,B). However, all the transgenic plants of Line 1 showed highly tolerance after spraying with 900 to 2700 g a.e.ha^–1^ glyphosate (Figures 3C–E). When the concentration of glyphosate was reached up to 3600 g a.e.ha^–1^, the transgenic plants of Line 1 still grew normally and showed no difference compared with water control (Figure 3F). HJ06-698 which only contained a glyphosate tolerant gene *CP4-EPSPS* also showed markedly tolerance to glyphosate but appeared “yellow flashing” in the young leaves, as the typical visual symptom of glyphosate application (Figure 3G), the duration of “yellow flashing” were positively correlated with the spraying doses of Roundup (Table 2). After spraying with Roundup at the rate of 900 g a.e.ha^–1^, the time of yellow flashing were continued up to seven to 10 days. However, this time could continue up to 1 to 2 months after spraying at the rate of 3600g a.e.ha^–1^.

**TABLE 2:** **Comparison of tolerance level for transgenic line 1 and HJ06-698**.

**GM soybeans**	**Rates of glyphosate (g a.e.ha^–1^)**	**Tolerance or not**	**Yellow flashing (Presence/Absence)**	**Duration (days)**
	900	Tolerance	Absence	–
Line 1	1800	Tolerance	Absence	–
	2700	Tolerance	Absence	–
	3600	Tolerance	Absence	–
	900	Tolerance	Presence	9 ± 2
HJ06-698	1800	Tolerance	Presence	17 ± 3
	2700	Tolerance	Presence	30 ± 5
	3600	Tolerance	Presence	45 ± 15

### Tissue and Glyphosate Induced Expression Analysis of Exogenous Gene

**FIGURE 4:**
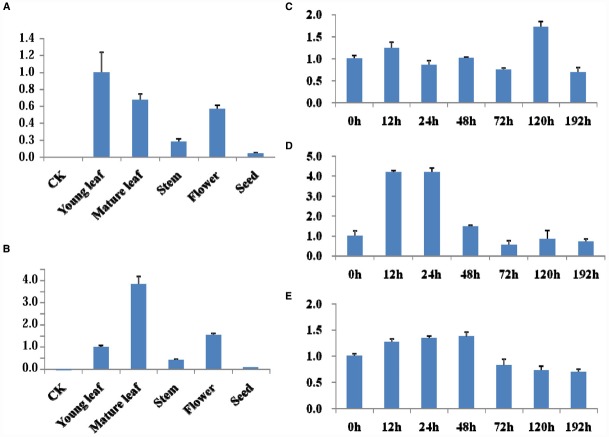
**mRNA level of the ***G2-EPSPS*** and ***GAT*** in transgenic line 1. (A–B)** qRT-PCR was used for analysis the expression level of *G2-EPSPS* gene **(A)** and *GAT* gene **(B)** in different tissues of transgenic soybean line 1. **(C–E)** qRT-PCR was used for analysis the expression level of *GAT* **(C)**, *G2-EPSPS* **(D)** in leaves of transgenic soybean line 1 and *CP4-EPSPS* **(E)** in leaves of HJ06-698 after treatment with 900 g a.e.ha^–1^ Roundup at different times.

qRT-PCR was used for analysis the target genes expression in different tissues (young leaves, mature leaves, stem, flower, and seed) of Line 1 without glyphosate application or leaves of Line 1 and HJ06-698 treated with 900 g a.e.ha^–1^ glyphosate at different time points. The highest expression level of target genes was detected in leaves while relative low expression level of transcript in seed and root (Figures 4A,B). After the treatment of glyphosate, no significant variation of transcript accumulation in leaves was detected at any time points when compared with control of no treatment (0 h; Figure 4C,D). Moreover, our results also suggested that the expression of *G2-EPSPS* in leaves of transgenic soybean Line 1 was consisted with that of *CP4-EPSPS* in HJ06-698 (Figure 4E), indicating similar functions of *G2-EPSPS* and *CP4-EPSPS* in these transgenic soybeans.

### Chlorophyll and Shikimic Acid Content Analysis of Transgenic Soybean

**FIGURE 5:**
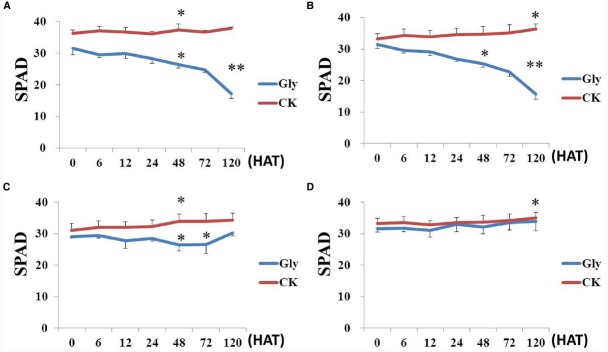
**Chlorophyll contents in transgenic plants.** Plants spraying with water were used as the negative control (CK) to analysis the chlorophyll content variation in different soybean cultivars after treatment of Roundup with the rate of 900 g a.e.ha^–1^ (Gly). **(A)** non-transgenic soybean MD12; **(B)** non-transgenic soybean ZH10; **(C)** GR1 transgenic soybean line HJ06-698; **(D)** transgenic soybean line 1. The difference between treatments has been shown as *P < 0.05 and **P < 0.01.

The chlorophyll contents of leaves in transgenic and non-transgenic soybeans were measured after spraying with labeled rate of Roundup. Compared with control, glyphosate significantly decreased chlorophyll content of non-transgenic soybeans including ZH10 and MD12 (Figures 5A,B). The chlorophyll content of HJ06-698 showed first decreased and then increased trends after spraying with Roundup (Figure 5C). This phenomenon is the results of transient “yellow flashing” in GR1 and GR2 transgenic soybeans. However, transgenic line 1 showed no decrease of chlorophyll content after spraying with Roundup and the trends was similar to water control (Figure 5D), which consistent with the result of no “yellow flashing” phenotype in this transgenic line.

**FIGURE 6:**
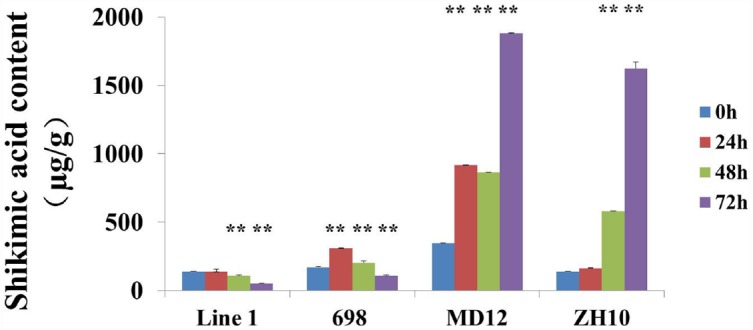
**Contents of the shikimic acid in transgenic soybean.** The contents of shikimic acid were measured in transgenic soybean line 1, HJ06-698, ZH10, and MD12 after treatment of Roundup with the rate of 900 g a.e.ha^–1^ for different times. The significance between different times has been shown as **P < 0.01.

Shikimic acid accumulation as a biomarker was usually used for evaluating the effect of glyphosate. In order to compare the accumulation of shikimic acid in transgenic soybeans, the content of shikimic acid was also measured after treatment with Roundup. The results showed that shikimic acid was significantly increased in non-transgenic plants ZH10 and MD12 after application of glyphosate (Figure 6). The accumulation of shikimic acid in HJ06-698 was extremely lower than that of non-transgenic control and showed first increased and then decreased trends. This result was consistent with the phenomenon of “yellow flashing” and the changes of chlorophyll content after treatment of glyphosate. However, accumulation of shikimic acid could not be detected in transgenic line 1 (Figure 6). These results indicated that the phytotoxicity of new transgenic soybean which co-expressed with *G2-EPSPS* and *GAT* genes had no or small effect on plant development after application of glyphosate.

## Discussion

At present, most commercial glyphosate tolerance soybeans utilized glyphosate tolerant genes (*CP4-EPSPS* and *2m EPSPS*) or glyphosate acetyltransferase gene (*GAT 4601*) separately (http://www.isaaa.org). *CP4-EPSPS* was derived from the soil bacterium *Agrobacterium* sp. Strain CP4 and the enzyme activity of CP4-EPSPS could not be inhibited by glyphosate due to weak binding affinity, resulting in increased tolerance to glyphosate in transgenic plants expressing this gene (Franz et al., 1997; Guicheney et al., 2009; Xiao et al., 2012). *GAT* encodes an N-acetyltransferase for acetylation of glyphosate, which is the basis of a new mechanism of glyphosate tolerance in GM plants (Castle et al., 2004; Siehl et al., 2005, 2007). Although *EPSPS* or *GAT* genes were widely used in commercial glyphosate tolerance crops alone, few researches focused on the new strategy to develop glyphosate tolerance plants by co-expression of these two kinds of genes. Our results suggested that high tolerant transgenic soybeans could be obtained by co-expression of *GAT* and *EPSPS*.

For GR1 and GR2 soybeans over-expressing *CP4-EPSPS*, the labeled rate for glyphosate application is varied from 600 to 1200 g a.e.ha^–1^ (Gazziero et al., 2008). Although the tolerant level could reach to even threefolds of labeled rate (2400 g a.e.ha^–1^) at some growth stages, the visual plant injury was often reported in glyphosate tolerant soybeans (Zobiole et al., 2012). In this study, transgenic soybean co-expressed with *G2-EPSPS* and *GAT* genes showed high tolerance to glyphosate and the tolerant level could reach to even fourfolds of labeled rate (3600 g a.e.ha^–1^) with no visual plant injury. Since the activity of EPSP synthase was inhibited by glyphosate and prevent the shikimic acid converted to EPSP, the accumulation of shikimic acid in the young leaves and other tissues of plants usually acts as a marker for evaluating the effect of glyphosate (Escorial et al., 2001; Pline et al., 2002; Shaner et al., 2005; Buehring et al., 2007; Henry et al., 2007). Generally, the more shikimic acid the plants accumulated, the more sensitive of the plants to glyphosate is. Therefore, no significant changes of shikimic acid content in transgenic plants co-expressed with *G2-EPSPS* and *GAT* genes after treatment with glyphosate resulted in the high tolerance of transgenic line 1 in this study.

Some reports also revealed that the typical symptom of “yellow flashing” and chlorophyll content decrease appeared in both GR1 and GR2 transgenic soybean after the application of glyphosate even at the labeled rate of 800 g a.e.ha^–1^ and other glyphosate tolerance plants (Mueller et al., 2003; Zobiole et al., 2010a, 2012). This phenomenon was resulted from the accumulation of primary phytotoxic metabolite (such as shikimic acid) in the plant meristems (Reddy et al., 2004; Ding et al., 2011). Moreover, the photosynthesis, nutrient accumulation, and nodulation in some cultivars of GR2 soybean was also reduced after receiving increasing glyphosate rates and application at later growth stages (Zobiole et al., 2012). In this study, the typical symptom was also observed in HJ06-698 derived from GR1 transgenic soybean even at labeled rate of Roundup (900 g a.e.ha^–1^). After spraying with Roundup, the chlorophyll content of leaves in HJ06-698 was first decreased and then increased (Figure 5). This result was consistent with the variation tendency of shikimic acid content in other glyphosate tolerance cultivars (Zobiole et al., 2011). These changes might be the direct explanation of transient “yellow flashing” in overexpressing *CP4-EPSPS* gene soybeans (Reddy and Zablotowicz, 2003; Zablotowicz and Reddy, 2007). The duration of “yellow flashing” in HJ06-698 was positively correlated with the spraying rates of Roundup (Table 2), which were similar to previous reports in corn and soybeans (Mahoney et al., 2014). Although “yellow flashing” is usually considered non-persistent because it tends to disappear within 2 weeks after the application of herbicide (Reddy and Zablotowicz, 2003), the decrease of chlorophyll content direct causes the reduction in photosynthesis and affects nutrient uptake and leads to lower plant biomass production and reduced grain yield (Zobiole et al., 2010b, 2012). In contrary, the typical symptom of glyphosate, such as accumulation of shikimic acid, “yellow flashing” and decreased chlorophyll content were not found in our transgenic plant after the treatment of glyphosate at different rates of 900–3600 g a.e.ha^–1^. This may due to the introduction of *GAT* besides of *EPSPS* results in reducing “true” concentration of glyphosate in soybean organs.

*G2-EPSPS* was identified from *Pseudomonas fluorescens* G2 isolated from a storage area with a history of glyphosate pollution and showed high glyphosate tolerance in transgenic tobacco and maize (Zhu et al., 2003; Dun et al., 2007; Liu et al., 2015). Dun et al. (2014) reported that the transgenic tobacco contained *G2-EPSPS/GAT* showed higher tolerance to glyphosate than tobacco contained only *G2-EPSPS* or *GAT* alone, and *G2-EPSPS* carrying tobaccos were significantly more susceptible to glyphosate than tobacco contained *GAT* only. The combination of the metabolic detoxification and target enzyme of glyphosate may result in high tolerance in transgenic crops, providing a new strategy for developing glyphosate tolerant crops. The introduction of detoxification genes like *GAT* can remove the glyphosate residue, resulting in more robust of tolerance. Moreover, it can also allow farmers to use glyphosate at any stage including reproductive stage of plant development (Pollegioni et al., 2011). In this study, the similar variation of expression level for *G2-EPSPS* and *CP4-EPSPS* genes after treatment of glyphosate indicated that these genes perhaps have similar function in transgenic plants. Therefore, the glyphosate detoxification function of *GAT* might be acts an important role in enhancing the glyphosate tolerance in GM crops.

## Author Contribution

LQ, YG, and BG conceived and designed the experiments; BG, YG, HH, LJ, and LZ performed the experiments; LQ, YG, BG, RC, WL, and ML analyzed and interpreted the data; LQ, BG, and YG drafted the paper. All authors read and approved the final manuscript.

### Conflict of Interest Statement

The authors declare that the research was conducted in the absence of any commercial or financial relationships that could be construed as a potential conflict of interest.

## Abbreviations

AMPA: Aminomethylphosphonic acid
AS: Acetosyringone
DTT: Dithiothreitol
EPSPS: 5-enolpyruvylshikimate-3-phosphate synthase
GAT: Glyphosate N-acetyltransferase
GOX: glyphosate oxidoreductase
GR1: First generation of glyphosate resistant crops
GR2: Second generation of glyphosate resistant crops
PVDF: Polyvinylidene difluoride
qRT-PCR: Quantitative real-time polymerase chain reaction.
